# Aberrant Fetal Brain Sulcus Formation: A Clue to the Diagnosis of Sotos Syndrome

**DOI:** 10.1002/pd.6686

**Published:** 2024-10-19

**Authors:** Caiqun Luo, Yang Liu, Hui Wang, LiYuan Chen, XiaoXia Wu, Qian Geng, Huaxuan Wen, Shengli Li, Weiqing Wu, Mei Zhong

**Affiliations:** ^1^ Department of Obstetrics and Gynecology Nanfang Hospital Southern Medical University Guangzhou China; ^2^ Maternal Fetal Medicine Center Shenzhen Maternity and Child Healthcare Hospital Southern Medical University Shenzhen China; ^3^ Medical Genetic Center Shenzhen Maternity and Child Healthcare Hospital Southern Medical University Shenzhen China; ^4^ Ultrasound Department Shenzhen Maternity and Child Healthcare Hospital Shenzhen China

## Abstract

**Objective:**

This study aims to elucidate two distinct fetal ultrasound features associated with aberrant brain sulcus formation as potential prenatal markers for Sotos syndrome caused by mutations in the NSD1 gene.

**Method:**

This retrospective study investigated three fetuses across two pregnancies, including a pair of monochorionic diamniotic twins, all diagnosed with Sotos syndrome via whole exome sequencing (WES). Comprehensive clinical and laboratory data were collected and analyzed. Each fetus underwent a series of specialized neurosonographic assessments to evaluate the development of the cerebral cortex.

**Results:**

All three fetuses exhibited aberrant brain sulcus formation characterized by Sylvian fissure (SF) abnormalities and shallow parietooccipital sulcus (POS). WES revealed the presence of two de novo NSD1 variants in these fetuses.

**Conclusions:**

Fetal aberrant brain sulcus formation may represent a distinctive ultrasound feature indicative of Sotos syndrome, thereby offering additional diagnostic insights for the identification of this condition.

**Summary**

- What’s already known about this topic?
  ◦ Prenatal ultrasound findings commonly seen include macrocephaly and mild cerebral ventricul‐omegaly in Sotos syndrome.
- What does this study add?
  ◦ We contribute an additional ultrasonographic phenotype, specifically aberrancy in brain sulcation, to aid in the prenatal diagnosis of Sotos syndrome.

## 1 Introduction

Sotos syndrome is caused by deletions or intragenic variants in NSD1 gene located on chromosome 5 [1]. Clinical manifestations encompass macrocephaly, tall stature, cardiac anomalies, excessive growth during childhood, distinctive facial appearance, various degrees of learning difficulties, and behavioral problems [2, 3]. Affected individuals also face an increased risk of neoplasms [4]. Few studies have investigated the fetal presentation of Sotos syndrome. Previous studies have reported abnormal postnatal neuroimaging findings in all patients with Sotos syndrome [5, 6], including enlargement of the lateral ventricles, trigones, and occipital horns, corpus callosum hypoplasia, enlarged cisterna magna, heterotopias, macrocerebellum, and periventricular leukomalacia. Fetal ultrasound findings in eight cases with Sotos syndrome suggest non‐specificity, with the most common finding being mild ventriculomegaly [7]. A recent 2024 case report described the nonspecific prenatal ultrasound features of Sotos syndrome as including ventricular dilatation, periventricular pseudocysts, and increased periventricular echogenicity [8].

We identified two unrelated families with three fetuses exhibiting aberrant brain sulcus formation. These fetuses were diagnosed with Sotos syndrome due to de novo mutations in NSD1 gene, as determined by whole‐exome sequencing (WES). The prenatal ultrasound finding of aberrant brain sulcus formation was previously unreported in Sotos syndrome. In our study, we describe and explore in detail the aberrant brain sulcus formation which we identified in all three cases and which appears to represent a prenatal finding characteristic of Sotos syndrome that should instigate a specific investigation for this condition.

## 2 Materials and Methods

### 2.1 Clinical Data

Two patients were referred for consultation at the Prenatal Diagnosis Centre of Shenzhen Maternity and Child Healthcare Hospital between October 2018 and October 2022.

Family 1: A 25‐year‐old gravida 2, para 1, had given birth to a healthy girl three years ago. She and her non‐consanguineous husband were referred for genetic counseling due to fetal growth restriction (EFW 0.4%) and small head circumference (−2.1SD) at 25 + 4 weeks in her second pregnancy. Maternal biochemical serological Down syndrome screening in the first trimester and Non‐Invasive Prenatal Screening (NIPS) for common fetal aneuploidy were negative. The patient elected to undergo amniocentesis.

Family 2: A 40‐year‐old healthy gravida 2, para 1, naturally conceived monochorionic diamniotic twins. She had given birth to a healthy girl by cesarean section a year ago. First trimester nuchal translucency screening sonograms were normal for both twins. Maternal biochemical serological screening of Down syndrome in the first trimester showed high risk. Amniocentesis was suggested to be performed because of the high risk of Down syndrome and advanced maternal age. She initially declined amniocentesis and elected NIPS, the result of which was low risk. Prenatal ultrasound had not found any abnormalities in her two fetuses before 23 weeks. At 24 + 6 weeks, one of the fetuses had mild cerebral ventriculomegaly (11.8 mm) for which the patient underwent amniocentesis on both fetuses.

### 2.2 Tertiary Level Ultrasound and Cortical Development Assessment

#### 2.2.1 Instruments

A Samsung A80 Doppler ultrasound scanner was used. Equipped with a 1–8 MHz single crystal volume probe, it has dynamic image storage and recall playback functions capable of post‐processing 2D and 3D images.

#### 2.2.2 Neurosonographic Methodology

For the assessment of cerebral cortical development, the Sylvian, parieto‐occipital, and calcarine fissures, as well as the cingulate sulcus and sulci over the cerebral convexity were evaluated, and the sonograms were evaluated by transabdominal ultrasonography. A detailed and defined method of cortical development assessment developed at our institution in the Ultrasound Department of Shenzhen Maternity and Child Healthcare Hospital was employed, as previously described [9, 10]. Using ultrasonography, the insula, Sylvian fissure (SF), parieto‐occipital sulcus (POS) and calcarine fissure (CF) were examined and compared to defined standard views of the fetal sulci as well as to the normal reference ranges of these sulcal measurements between 18 and 41 weeks of gestation. During normal development, the SF width, temporal lobe depth, POS depth, and the CF depth increase with advancing gestation. The width of the uncovered insula and the POS angle decrease with advancing gestation. Using the aforementioned published reference data, brain sulcus development is considered aberrant if the appropriate cranial ultrasound images have been obtained but one or more of the aforementioned sulci or fissures were either not visualized at the expected gestational age, or their appearance was abnormal for the gestational age. We employed this standardized neurosonography protocol inclusive of our established morphology classification of abnormal SF [11] in the evaluation of all three fetuses in the current case series.

#### 2.2.3 Trio‐WES

The three fetuses underwent Trio‐WES (father‐mother‐fetus) following normal or non‐diagnostic karyotype and chromosomal microarray (CMA) results. Fetal DNA was extracted from amniotic fluid, and parental DNA was extracted from peripheral blood samples. Both parents provided written informed consent for the clinical WES after receiving an explanation regarding the benefits and limitations of the test. WES testing and interpretation were performed in the medical genetics laboratory of SHEN ZHEN Maternal and Child Healthcare Hospital. Genomic DNA from blood and amniotic fluid was extracted using the QIAGEN Blood Mini Kit. The Berry Nano WES chip was used to capture and enrich the DNA sequence in exons and flanking intron sequences of approximately 20,000 genes. The Illumina NovaSeq platform was used for sequencing. Bioinformatics analysis was conducted using SeqMax software. Disease‐causing variants were identified using a pipeline created by our laboratory. Variant classification was performed according to the ACMG guidelines [12]. Pathogenic (P) and likely pathogenic (LP) variants that could explain the fetal phenotypes were reported. Variants of uncertain significance (VUS) were discussed by a multidisciplinary team to decide whether to report them or not. All pathogenic and likely pathogenic variants detected by WES were confirmed by Sanger sequencing.

## 3 Results

### 3.1 Family 1

**FIGURE 1:**
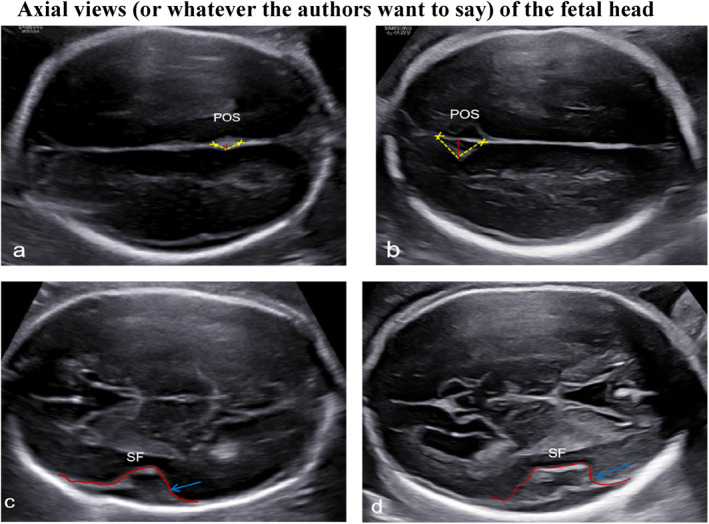
Ultrasound images of the Sylvian fissure (SF) and parieto‐occipital sulcus (POS) at 25 weeks in family 1 fetus compared to a fetus with a normal neurological system (The yellow dotted line indicates the angle of the parieto‐occipital sulcus [POS], the red dotted line indicates the depth of the parieto‐occipital sulcus [POS], and the red solid line indicates the shape of the Sylvian fissure [SF]. The same applies below.) (a) The POS of family 1 fetus at 25 weeks. (b) The POS of a fetus with a normal neurological system at 25 weeks. The POS depth of the fetus in family 1 is significantly lower than that of normal fetuses at 25 weeks (c) The SF of family 1 fetus at 25 weeks. (d) The SF of a fetus with a normal neurological system at 25 weeks.

**FIGURE 2:**
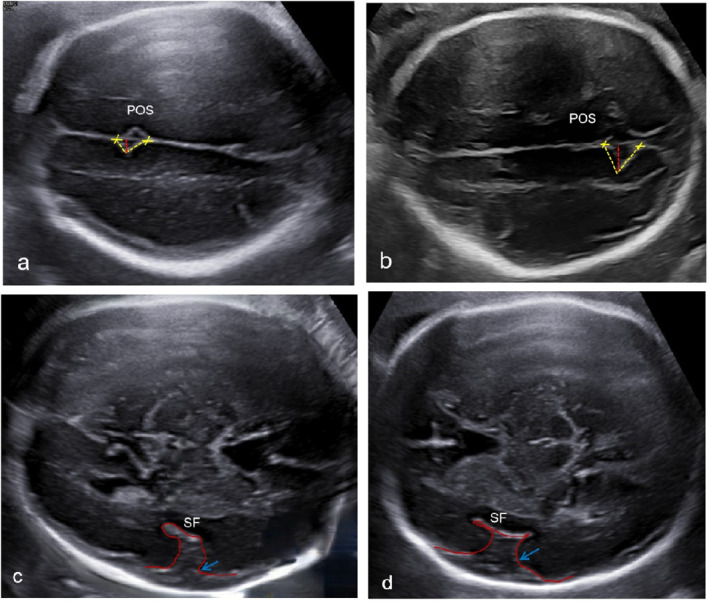
Ultrasound images of the Sylvian fissure (SF) and parieto‐occipital sulcus (POS) at 29 weeks in family 1 fetus compared to a fetus with a normal neurological system. (a) The POS of family 1 fetus at 29 weeks. (b) The POS of a fetus with a normal neurological system at 29 weeks. The POS depth of the fetus in family 1 is significantly lower than that of normal fetuses at 29 weeks (c) The SF of family 1 fetus at 29 weeks. (d) The SF of a fetus with a normal neurological system at 29 weeks.

Fetal sonogram in our center at 25 + 4 weeks showed abdominal circumference less than the 10th percentile, and small head circumference (−2.1SD) with no extracranial structural anomalies. The fetal parieto‐occipital sulcus (POS) depth was found to be less, and the POS angle was increased (Figure 1a) compared to fetuses with a normal neurosonographic examination at 25 weeks (Figure 1b). The shape of the Sylvian fissure (SF) was also abnormally developed for gestational age (Figure 1c) as compared to normal (Figure 1d). The fetal brain sulcus formation was considered aberrant. At 29 + 5 weeks, the head circumference of the fetus was normal (−0.83sd), and fetal growth restriction was not found; however, the POS depth was less and the POS angle was increased (Figure 2a) compared to fetuses with a normal neurosonographic examination at 29 weeks (Figure 2b), The SF was also abnormal for gestational age (Figure 2c) as compared to normal (Figure 2d). A pathogenic variant of NM_022455.4 c.6115C>T (p.Arg2039Cys) in the NSD1 gene was identified by trio‐WES. The parents did not have the NSD1 variant. The fetus was diagnosed with Sotos syndrome. After being informed of the diagnosis, the couple decided to carry the pregnancy to term. At 37 + 6 weeks, the pregnant woman gave birth to a boy via vaginal delivery; the birth weight was 2450 g with normal Apgar scores of 10 at 1 and 5 min. The head circumference at birth was 33.1 cm (0.3sd). The baby had an unremarkable facial appearance at birth but later displayed delayed milestones and developed macrocephaly, frontal bossing, a pointed chin, large hands, and expressive language delay at the 26‐month follow‐up.

### 3.2 Family 2

**FIGURE 3:**
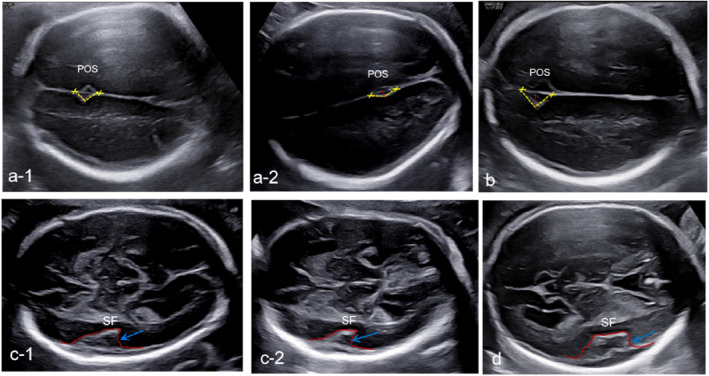
Ultrasound images of the Sylvian fissure (SF) and parieto‐occipital sulcus (POS) at 25 weeks in family 2 fetus compared to a fetus with a normal neurological system. (a‐1, a‐2) The POS of two fetuses in familily 2 at 25 weeks. (b) The POS of a fetus with a normal neurological system at 25 weeks. The POS depth of the two fetuses in family 2 are significantly lower than that of normal fetuses at 25 weeks (c‐1, c‐2) The SF of two fetuses in familily 2 at 25 weeks. (d) The SF of a fetus with a normal neurological system at 25 weeks.

At 25 + 6 weeks, the ultrasound found the fetal POS depth to be less and the POS angle increased (Figure 3a‐1,a‐2) compared to fetuses with a normal neurological system (Figure 3b), and the shape of the SF was also abnormal for gestational age (Figure 3c‐1,c‐2) as compared to normal fetus at 25 weeks (Figure 3d), indicating aberrant brain sulcus formation for the two fetuses. The size of the fetuses was appropriate for gestational age, and the lateral ventricle of one fetus was enlarged (12.6 mm). The two fetuses had the same Short Tandem Repeat (STR) by QF‐PCR and Trio‐WES identified a de novo likely pathogenic variant of NM_022455.5: c.971G>T (p.Gly324Val) in NSD1 gene. Parental studies were negative. Following detailed genetic and multidisciplinary counseling, the couple elected to carry the pregnancy to term.

## 4 Discussion

Sotos syndrome is estimated to have a prevalence of one in 15,000 live births [1], and is often diagnosed in the first 2–3 years of life when cardinal features appear, including excessive growth during childhood, macrocephaly, characteristic facial appearance and various degrees of learning difficulty, along with variable minor features. Few reports have investigated Soto syndrome in terms of its fetal presentation. Previously described prenatal features of Sotos syndrome include: increased nuchal translucency in the first trimester, mild cerebral ventriculomegaly in the second trimester, and polyhydramnios and macrocephaly in the third trimester. Two fetuses with Sotos syndrome had a high risk on Maternal biochemical serological Down syndrome screening in the first trimester, and fetal growth restriction (FGR) and renal abnormalities have also been reported. No sonographic finding common to all affected fetuses has been described [7, 13, 14, 15, 16, 17].

Cerebral gyral and sulcal formation is viewed as a fundamentally important developmental process of the early fetal brain. Visible aberrations to the shape of the gyral folds and the length or depth of their associated fissures are considered to be signs of fetal brain maldevelopment. Delayed or absent cerebral sulcation is likewise considered a principal feature of fetal malformations in cortical development (MCD) with described abnormalities that include premature abnormal sulci, thin and irregular cortical mantle, wide, abnormally overdeveloped gyri, wide opening of isolated sulci, nodular bulging into the lateral ventricles, cortical clefts, and intraparenchymal echogenic nodules [18, 19, 20]. Additional ultrasound signs suggestive of MCD include delayed cortical development, dysgenesis of the Sylvian fissure, delayed sulcal appearance, cortical thickening, irregularity of the ventricular wall, absence or abnormal appearance of fissures, abnormal, asymmetric gyri, and discontinuous cortex [21, 22]. MCD can affect neurodevelopmental outcome in multiple ways, including cognitive disability, schizophrenia, and poor coordination [23, 24].

A fetal cerebral cortex assessment is indicated particularly when the fetal head circumference is small and there are other central nervous system (CNS) abnormalities. The CNS is not fully mature until childhood, and at least part of the dynamic process that takes place in the developing brain can be appreciated by fetal imaging. The pattern of normal fetal cerebral cortical development, however, especially the appearance of the cerebral fissures and sulci prior to 30 weeks of gestation, is not familiar to many sonographers, and the assessment of the developing fetal brain can be challenging, even for experienced sonographers. Recent studies to better define the development of the cerebral sulci and gyri by measuring the parietal–occipital fissure (POF) depth, POF angle, sylvian fissure (SF) depth, SF width, uncovered insular width, calcarine fissure (CF) depth, hemisphere depth on the views of POF, SF, and CF, uncovered insular ratio, biparietal diameter (BPD) and head circumference (HC) have provided opportunities to improve our understanding of the developing fetal brain [9, 10, 11].

In the current study, we observed that three fetuses with Sotos syndrome, who had de novo genetic variants in the NSD1 gene, exhibited aberrant brain sulcus formation in the second and third trimesters. The depth of the fetal parieto‐occipital sulcus (POS) was reduced, and the POS angle was larger compared with that of fetuses with a normal neurological system at the same gestational age. The Sylvian fissures (SF) displayed an abnormal shape, a finding not previously reported. One limitation of our study is that the measurements of the Sylvian fissure (SF) and parieto‐occipital sulcus (POS) in fetuses cannot currently be translated into specific numerical values for further quantitative analysis and comparison.

Prior research [25] indicated that the NSD1 gene is located in neural gene regions targeted by PAX6/PAUPAR due to its binding to the C‐terminus of PAX6. The interaction between PAX6 and NSD1 plays an important role in maintaining the H3K36me3 modification at these neural gene regions and in the cortical differentiation of human embryonic stem cells (hESCs), and the PAUPAR/PAX6/NSD1 complex plays a critical role in the epigenetic regulation of hESC brain cortical differentiation. We speculate that mutations in the NSD1 gene lead to abnormal cerebral cortical development. This may manifest as specific prenatal ultrasound signs of Sotos syndrome, and our study suggests that the SF and POS could serve as potential ultrasound markers for its diagnosis. However, further studies with a greater sample size and further mechanistic research are required to confirm these discoveries.

## Ethics Statement

The authors have nothing to report.

## Consent

Written consent was obtained from each patient in this study for participation in this research.

## Conflicts of Interest

The authors declare no conflicts of interest.

## Data Availability

The data that support the findings of this study are available on request from the corresponding author. The data are not publicly available due to privacy or ethical restrictions.
